# "She would sit with me": mothers' experiences of individual peer support for exclusive breastfeeding in Uganda

**DOI:** 10.1186/1746-4358-5-16

**Published:** 2010-10-26

**Authors:** Jolly Nankunda, James K Tumwine, Victoria Nankabirwa, Thorkild Tylleskär

**Affiliations:** 1 Department of Paediatrics and Child Health, Makerere University College of Health Sciences, P.O Box 7072, Kampala, Uganda; 2 Centre for International Health, University of Bergen, Norway

## Abstract

**Background:**

Different strategies have been used to improve the initiation and duration of breastfeeding. Peer counsellors are reported to improve exclusive breastfeeding levels, but few studies have assessed the satisfaction of women with the support given, especially in Africa. In this paper we describe women's experiences of peer counselling for exclusive breastfeeding in an East African setting.

**Methods:**

In the Ugandan site of PROMISE-EBF, a multi-centre community randomised trial to evaluate the effect of peer counselling for exclusive breastfeeding on infant health, 370 women in the intervention arm participated in a study exit interview. Individual peer counselling was offered to women in 12 of the 24 study clusters, scheduled as five visits: before childbirth and during weeks 1, 4, 7 and 10 after childbirth. During the visits, the women were given information and skills to help them breastfeed exclusively. After the 10-week visit, they were interviewed about their feelings and experiences related to the peer counselling.

**Results:**

Overall, more than 95% of the women expressed satisfaction with the various aspects of peer counselling offered. Those who had received five or more visits were more likely to give positive responses about their experience with peer counselling than those who had received fewer visits. They explained their satisfaction with time spent with the peer counsellor in terms of how much she discussed with them. Most women felt their knowledge needs about breastfeeding were covered by the peer counsellors, while others expressed a desire to learn about complementary feeding and family planning. Attributes of the peer counsellors included their friendliness, being women and giving support in a familiar and relaxed way. Women were positive about the acquisition of knowledge and the benefit to their babies from the peer counselling. They preferred a peer counsellor to a health worker for support of exclusive breastfeeding because of their friendly approach.

**Conclusions:**

Individual peer counselling to support exclusive breastfeeding was positively received by the women.

**Trial Registration:**

clinicaltrials.gov no: NCT00397150.

## Background

The critical importance of exclusive breastfeeding for child survival has continued to be highlighted [1,2]. In Africa, almost all mothers breastfeed their infants, but exclusive breastfeeding for the first six months of life is rare [3-6]. In the recent Maternal and Child Under-nutrition *Lancet *series, it was reported that in Africa only 47% of infants younger than two months are exclusively breastfed [7]. This was reported to drop to 25% in those aged 2-5 months, and that 6% of children aged 6-11 months had stopped breastfeeding [7].

The widespread use of prelacteal feeds, as well as early introduction of complementary foods with low energy density before the recommended six months, has been reported in a number of sub-Saharan countries including Uganda [3-6,8-10]. In addition, the risk of HIV transmission from mother to infant through breast milk is twice as high with mixed feeding as it is with exclusive breastfeeding [11-14]. Therefore, there is a continued need to search for practical, acceptable and affordable ways to help mothers to breastfeed exclusively in the African setting, not only among the large majority of mothers who are not HIV infected or have unknown status, but also among HIV infected mothers living in situations where replacement feeding is not affordable, feasible, acceptable, sustainable or safe (AFASS) [15]. Widespread societal support for exclusive breastfeeding is a prerequisite for HIV-infected women not to be stigmatised by rigorous application of exclusive breastfeeding in non-AFASS situations.

In Mbale District in Eastern Uganda, breastfeeding is almost universal among women, but exclusive breastfeeding is not commonly practised [6]. The use of prelacteal feeds is widespread, with more than 50% of babies receiving them. The most common reasons given for this practice were waiting for the breast milk to come in, satisfying the baby's hunger, cleaning the baby's throat or allowing the mother to recover from the pain and exhaustion of delivery [6].

It has been reported that improved levels of exclusive breastfeeding are supported by peer counselling and that supported mothers appreciate the benefits of the approach [16-23]. Most of these peer support interventions have been carried out in high income or Asian settings [16,18,19,21,22,24-27] with very little experience reported from the African setting. In many of these interventions, contact between the mother and peer counsellor has been by telephone, with minimal face-to-face interaction.

The use of peer counsellors to support exclusive breastfeeding presents a potentially acceptable and practical way to help mothers to breastfeed exclusively. However, few studies have evaluated the perceptions and feelings of supported mothers about peer support. The few that have explored this aspect have reported positive attitudes [28,29]. Many mothers supported by breastfeeding peers reportedly valued the social support provided by the peer counsellors highly, in addition to the practical or technical support and information they received [30]. They appreciated the friendly, non-dogmatic and non-didactic approach used by peer counsellors [30].

In this paper, we describe the experiences of the supported mothers towards individual peer counselling for exclusive breastfeeding in an African setting. We report on data from a sub-study done at the Uganda site of the PROMISE-EBF study, using both quantitative and qualitative information. PROMISE-EBF is an acronym for: *Promoting Infant Health and Nutrition in Sub-Saharan Africa: Safety and Efficacy of Exclusive Breastfeeding Promotion in the Era of HIV*. It is a multi-centre, community-randomised controlled trial in French speaking Burkina Faso in West Africa, Uganda in East Africa, Zambia in Central Africa and South Africa, with the major objective of assessing the impact of peer counselling for exclusive breastfeeding on exclusive breastfeeding rates and on child health in Africa (clinicaltrials.gov no: NCT00397150). The main study outcomes will be reported elsewhere.

## Methods

### Study site

The Uganda site for the PROMISE-EBF study is Mbale District, Eastern Uganda, about 230 kilometres from the Ugandan capital, Kampala. The study, carried out from September 2005 to June 2008, was undertaken in two of the seven administrative units of the district called counties: Mbale Municipality, and the rural Bungokho County. Mbale Municipality houses the district administrative offices and has approximately 10% of the district population [31] of about 720,000. It is surrounded by Bungokho County. Most of the population are subsistence farmers who predominantly grow food crops for home consumption, but sell the surplus in the nearby markets and trading centres. A few cash crops such as coffee and cotton are grown on a small scale.

A number of people have migrated to Mbale Municipality from the surrounding areas in search of work and better living conditions. Most of these, with minimal or no formal education or specific skills, have settled in the informal settlement areas of the municipality where overcrowding is rife. Many of them provide manual labour in the few factories within the municipality or do petty jobs in the business section, while some carry out petty trade activities for survival within these informal settlements. The majority belong to the Bagisu ethnic group who use Lumasaba as their main language, but there are minority ethnic groups - Iteso, Baganda and Bagweri - who speak different languages, but are usually able to understand Lumasaba.

### Study design

The Ugandan part of the PROMISE-EBF study was carried out in twenty-four clusters drawn from the two counties. Each cluster consisted of one to three villages, depending on the village population size. Each cluster had an estimated population of around 1000, so given a birth rate of 3.5% the expected number of babies born per year in each cluster was 35. The 24 clusters were randomised using community-randomisation. The clusters were stratified into either rural (18) or urban (6). In each stratum, they were randomly allocated to the intervention (peer counselling for exclusive breastfeeding) or control clusters. The intervention was subsequently set up in the twelve allocated clusters, nine rural and three urban. Two of the three urban clusters comprised densely populated settlement areas of Mbale Municipality with poor housing and overcrowding. The other twelve clusters acted as controls with no peer counsellors. Being a community-randomised study, all eligible women in a cluster were, by definition, randomised to either intervention or control, depending on where they lived.

In each cluster the study team used the (female) leader in charge of women and child affairs to identify pregnant women for possible recruitment into the study. They were referred to as "recruiters" for the study. On identifying a pregnant woman, the recruiter reported to the study office, which then made arrangements for a research assistant to visit the identified woman. The aim of this initial visit was to explain the purpose of the study, obtain written informed consent and to check eligibility. In the control clusters a total of six women declined participation in the PROMISE-EBF study. Eligible women were recruited into the study when they were about seven months pregnant or within the first week after delivery. To be eligible a woman had to be pregnant with no intentions of leaving the study area for at least two years and had to be planning to breastfeed the baby.

This paper. used both qualitative and quantitative data collection methods, an approach dubbed 'mixed methods' design and increasingly being used in health and behavioural research [32,33]. Women were interviewed after completion of the peer counsellors' visit schedules regarding their experiences with the different aspects of peer counselling. Interviews were semi-structured, using both open-ended and closed-ended questions.

### The peer counselling intervention

Twelve women, one from each cluster and resident in the intervention clusters, were selected as peer counsellors by village meetings with guidance from the study team. They were trained for six days using the WHO breastfeeding counselling course, modified to suit the peer counsellors' level of education. They were aged between 25 and 40 years and all except one had previous breastfeeding experience. The selection, training and follow-up of these peer counsellors and their characteristics have been described previously [34].

The intervention involved peer counsellors visiting the identified women in their own homes and having one-to-one sessions in which different aspects of breastfeeding were discussed. The women were however allowed to invite their husbands, mothers-in-law or mothers to participate in the sessions if they so wished. Each woman was scheduled to receive a minimum of five visits as follows: during pregnancy at around seven months and during weeks one, four, seven and ten following childbirth. However, if a woman needed extra visits for any reason, the peer counsellor was free to follow up and record them as extra visits. Two supervisors regularly supervised the peer counsellors.

### Subjects

**Figure 1:**
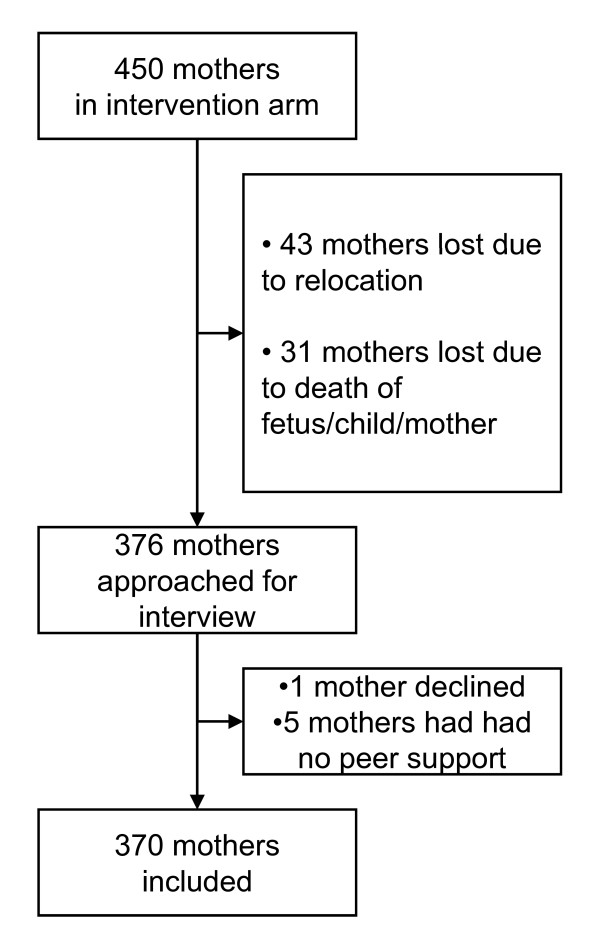
**Study profile. **Intervention arm of PROMISE-EBF trial

Of the 450 women recruited for the peer counselling intervention, 376 were approached for "exit" interviews after the completion of the peer support and the data collection for the main outcome (Figure 1). The remaining 74 were not interviewed because of relocation out of the study area, infant death, stillbirths, miscarriages, or maternal deaths. Out of the 376 approached, five reported that they were not visited by the peer counsellors so the interviews could not be completed, while one declined to complete the interview. A total of 370 interviews were conducted (Figure 1).

### Exit interviews

When the planned peer counsellor visits were completed, each woman was interviewed. The women's experience with the peer counselling process as well as some specific aspects such as the adequacy of time spent with the peer counsellor and usefulness of visits were assessed. This was done using a semi-structured questionnaire with both closed and open-ended questions. For the open-ended questions, the women's responses were recorded verbatim in the space provided for that purpose on the questionnaires.

Most of the interviews were conducted by a research assistant who was fluent in the commonly used local language, and the corresponding author observed the process and the women's non-verbal responses during some of the interviews. The research assistant was first trained in the study procedures regarding these interviews and was supervised by the corresponding author. The corresponding author also conducted some interviews assisted by an interpreter. This interaction helped to contextualise the women's responses and proved helpful during the process of coding the semi-structured responses.

The interviews were conducted at the mothers' homes. For logistical reasons they were not done at the same point in time after childbirth for all the participating women, but were done between 10 and 24 weeks following childbirth. Women were approached for interviews soon after completing the peer counsellor visit schedule. If a woman was not found at home for any reason, the research assistant kept going back until she found the woman or confirmed that the woman would not return within the study period. The interviews were conducted between August 2006 and April 2008.

### Ethical considerations

Permission to conduct the study was granted by Makerere University College of Health Sciences and by the Uganda National Council for Science and Technology. Written informed consent from the mothers was obtained in Lumasaba, the commonly used local language in the study area. The study used numbers for identification of participants and the responses of the women were kept confidential.

### Data management and statistical analysis

Responses to open-ended questions were post-coded by the research team. The coding of the women's responses to the semi-structured questions was done by four members of the study team throughout the study period. At each of the coding session, a questionnaire was picked by one team member, identified by its unique number which was recorded by each of the team members in their coding booklets. The member then read out the responses to the semi-structured questions without referring to the background information of the woman. Each member of the coding team contributed ideas they picked from the response and these were discussed and agreed on by the team. The team gave each idea a code and it was recorded in each member's booklet. In case of not understanding the read out response, the same response was read out by a different team member and consensus was reached by all members on whether all ideas in the response had been captured. Similar ideas were grouped together and themes emerging from these were identified. The team discussed these ideas and reached consensus about the emerging experiences of the women toward the different aspects of the peer counselling process. Themes that were dissimilar were resolved by consensus.

The women's experiences relating to the different aspects of peer counselling were categorised under the following pre-defined themes: satisfaction with explanations by peer counsellors, usefulness of visits by peer counsellor, interaction of peer counsellor with the women and women's suggestions regarding future peer counselling. Some sub-themes that emerged during the process - for example, taking time and not being in a hurry, and peer as teacher - are presented under the main themes. Selections of the mothers' responses to some questions are presented as verbatim quotations in this paper. The first author was a member of the coding team but the other members of the team were not actively involved in supervision of the peer counselling intervention.

The quantitative data from the interviews were entered using EpiData 3.1.software and were analysed using SPSS software, Version 15.

The women were asked to rate their satisfaction regarding the different aspects of the peer counselling process using a Likert-type, four or five point scale (1 - strongly disagree to 5 - strongly agree). The five items used for the scale were: time spent with peer counsellor, mother's satisfaction with peer counsellor's explanations, usefulness of visits to the mother, whether the mother felt free to talk to the peer counsellor and whether the mother felt that she was given a chance to ask questions. The 'time spent with peer counsellor' and 'mother given chance to ask questions' items had four scale points, each corresponding to the four alternatives given to the mother during the interview. The remaining three items had five points each, making a total of 23 points on the scale for the five items. An overall satisfaction score was generated from the responses using this combined score. A score of 70% or above was taken as overall satisfaction with the peer counselling. This corresponded to a woman scoring a total of 16 points on the five items and was used as the cut-off between satisfied and not satisfied with peer counselling overall. Overall satisfaction was the main outcome of this sub-study. The rest of the questions assessing other aspects of the peer counselling were analysed individually.

Categorical data were analysed using the Chi square test or Fischer's exact test for association between the women's characteristics and their satisfaction with the peer counselling. Continuous variables were summarised as means. The results obtained from analysis of the quantitative and qualitative data are presented together.

## Results

### Socio-demographic characteristics of the study women

The 370 women were aged 15 to 46 years with a mean age (SD) of 26 (6) years. The rest of the demographic characteristics of the mothers and their feelings about different aspects of peer support are summarised in Table 1. There was no significant association between the women's socio-demographic characteristics and their satisfaction with peer support except their education status (Table 1). The women who had completed primary or above were more likely to express satisfaction with peer support than those with incomplete primary or no education (Table 1). The number of women in each cluster ranged from 24 to 39 with an average of 31.

**Table 1:** Women's characteristics and their satisfaction with peer support (Percentages based on row total)

	Total	**Enough time with mother**^**a**^	**Mother satisfied with explanations**^**b**^	**Visits useful**^**c**^	**Learnt new thing**^**d**^	**Free to discuss**^**e**^	**Chance to ask questions**^**f**^	**Mother respected**^**g**^	**PC for next lactation**^**h**^	**PC for friend**^**i**^	**PC best to help with B/F**^**j**^	**Overall satisfaction**^**k**^	p-value
	N	n (%)*	n (%)*	n (%)*	n (%)*	n (%)*	n (%)*	n (%)*	n (%)*	n (%)*	n (%)*	n (%)*	
Total	370	286 (77)	331 (90)	358 (97)	350 (95)	350 (95)	265 (72)	365 (99)	361 (98)	365 (99)	263 (71)	264 (71)	
Age													
20 years and below	90	65 (72)	80 (89)	88 (98)	87 (97)	84 (93)	60 (67)	89 (89)	89 (99)	90 (100)	67 (74)	62 (69)	
More than 20 years	280	221 (79)	251 (90)	270 (96)	263 (94)	266 (95)	205 (73)	276 (99)	272 (97)	275 (98)	196 (70)	202 (72)	0.55
Education level													
None/Incomplete primary	90	64 (71)	75 (83)	82 (91)	87 (97)	82 (91)	63 (70)	88 (98)	87 (97)	88 (98)	66 (73)	55 (61)	
Completed primary	181	147 (81)	167 (92)	179 (99)	174 (96)	175 (97)	138 (76)	180 (99)	175 (97)	178 (98)	132 (73)	136 (75)	
Secondary and above	99	75 (76)	89 (90)	97 (98)	89 (90)	93 (94)	64 (65)	97 (98)	99 (100)	99 (100)	65 (66)	73 (74)	0.046
Marital status													
Single/other	22	13 (59)	21 (95)	21 (95)	21 (95)	22 (100)	16 (73)	22 (100)	22 (100)	22 (100)	19 (86)	16 (73)	
Married/cohabiting	348	273 (78)	310 (89)	337 (97)	329 (95)	328 (94)	249 (72)	343 (99)	339 (97)	343 (99)	244 (70)	248 (71)	0.88
Occupation													
Housewife	76	60 (79)	69 (91)	73 (96)	68 (90)	73 (96)	52 (68)	73 (96)	72 (95)	74 (97)	48 (63)	52 (68)	
Farmer	231	178 (77)	202 (87)	225 (97)	224 (97)	217 (94)	164 (71)	229 (99)	228 (99)	228 (99)	177 (77)	167 (72)	
Other	63	48 (76)	60 (95)	60 (95)	58 (92)	60 (95)	49 (78)	63 (100)	61 (97)	63 (100)	38 (60)	45 (71)	0.81
Parity													
One child	67	49 (73)	60 (90)	66 (99)	64 (96)	62 (92)	45 (67)	66 (99)	66 (99)	67 (100)	47 (70)	48 (72)	
Two or more children	303	237 (78)	271 (89)	292 (96)	286 (94)	288 (95)	220 (73)	299 (99)	295 (97)	298 (98)	216 (71)	216 (71)	0.95
No of peer counsellor visits													
5 or more	212	177 (84)	198 (93)	209 (99)	207 (98)	205 (97)	171 (81)	208 (98)	211 (99.5)	209 (99)	167 (79)	170 (80)	
Less than 5	158	109 (69)	133 (84)	149 (94)	143 (91)	145 (92)	94 (60)	157 (99)	150 (95)	156 (99)	96 (61)	94 (60)	<0.01

p-values (Chi square test) reflect association between overall satisfaction of the mothers and their characteristics.

^a^- Mother felt peer counsellor spent enough time with her during visits

^b^- Mother was satisfied with peer counsellor's explanations about breastfeeding

^c^- Mother felt peer counsellor's visits were useful to her

^d^- Mother learnt something new from the peer counsellor

^e^- Mother felt free to discuss with the peer counsellor

^f^- Mother felt peer counsellor gave her chance to ask questions

^g^- Mother felt peer counsellor treated her with respect during visits

^h^- Mother would welcome a peer counsellor for her next pregnancy

^i^- Mother would recommend a peer counsellor for a friend

^j^- Mother feels peer counsellor is the best to help women with breastfeeding

^k^- Overall satisfaction of the mother

*- Percentages are based on the total number of mothers in the rows which appears in the first column of each row.

Overall, the women (71%) were satisfied with the peer counselling they received (Table 1). More than 95% expressed satisfaction regarding the following aspects: usefulness of the visits, having learnt something new about breastfeeding, having felt free to discuss with the peer counsellor during visits and having felt respected by the peer counsellor during visits (Table 1). Almost all the women (99%) said they would welcome a peer counsellor again for the next pregnancy or recommend one for a friend (Table 1). Regarding the women's feelings about being given the opportunity to ask questions during visits, 72% felt the peer counsellor had given them enough opportunity to ask while 77% felt the peer counsellor spent enough time with them at each visit (Table 1).

### Number of peer counsellor visits and satisfaction

The number of visits received from the peer counsellors as reported by the mothers ranged from one to about 20 with a mean of five and a median of five. More than half the women (57.3%) reported having received five or more visits from the peer counsellor, while five women reported that they were not visited at all (Figure 1).

The number of visits the women received from the peer counsellor showed a significant association with their satisfaction with the peer counselling on univariate analysis. The women who received five or more visits were more likely to give positive responses regarding their satisfaction with the different aspects of peer counselling and were more likely to show overall satisfaction (Table 1).

### Spending enough time

The majority of the women (77%) said that they felt the peer counsellor had spent enough time with them during the visits (Table 1). The most commonly cited reasons were that the peer counsellor taught them a lot about breastfeeding, took time to explain and repeated whatever was not understood. In addition, the peer counsellor reportedly gave the women an opportunity to ask questions and asked them questions to check their understanding (Table 2).

**Table 2:** Women's feelings about time spent with peer counsellor (n = 370, multiple responses allowed for each woman)

Reasons for women's satisfaction with time spent *	n	%
Peer counsellor taught me a lot about breastfeeding	203	55

She took time to explain and repeated whatever I did not understand	107	29

She gave me chance to ask questions and also asked me questions	12	3

She taught me good things and how to keep my baby	7	2

Others	7	2

**Reasons for dissatisfaction**		

Peer counsellor was in a hurry and spent a short time	31	8

Peer counsellor came when I was busy with other things to do	7	2

I only learnt a few things from her	6	2

She only came to tell me to breastfeed my baby until six months	2	1

*The total number of reasons is not equal to the number of women interviewed because some women gave more than one reason while others gave none

Some of the women emphasised that the peer counsellors did not show that they were in a hurry to leave. Taking time with a mother and not appearing to be in a hurry during the visits was repeatedly echoed by the women as a way of describing enough time. A 20-year-old mother of one child said:

*"...when the peer counsellor comes we talk about everything till we finish. She would not be in a hurry. She would teach me and I understood"*.

Some women related "enough time" to the number of hours spent. A 16-year-old first time mother said:

*"...she could come at around four in the afternoon and leave at six o'clock, all the while teaching me how to breastfeed my baby and what I am to eat when breastfeeding"*.

Some women referred to being able to ask the peer counsellor questions in case they had not understood. A 28-year-old mother of four children said:

"...she would take long explaining to me about breastfeeding and I could also ask her a question when I had not understood and she repeated for me".

However, a few women reported that they were not satisfied with the time spent by the peer counsellor: the peer counsellor was in a hurry to leave and spent a short time, the peer counsellor visited at a time when the woman was busy with her household chores, and the woman learned very few things from the peer counsellor (Table 2).

Some women emphasised the timing of the visit and were concerned that the peer counsellors turned up at a time that was not convenient. A 29-year-old mother of five children said:

*"...sometimes the peer counsellor would come late or around lunch time when I would be trying to prepare lunch and we could not have much time together"*.

### Providing relevant knowledge and explanations

The large majority of the women (90%) were satisfied with the peer counsellors' explanations regarding exclusive breastfeeding (Table 1). They gave various reasons for their responses. The main ones were that the peer counsellor explained the importance of exclusive breastfeeding up to six months in such a way that they were able to understand and could remember. They also said the peer counsellor showed them how to assist their babies to latch on to the breast properly and gave them advice about breastfeeding that they perceived as relevant in their situation. They were satisfied with the knowledge and skills they acquired from the peer counsellors, and because the knowledge was given in a form and a language familiar to them, they were able to scrutinise it and decide whether it was reasonable. Many women viewed the peer counsellor as a "teacher" who taught them a lot about breastfeeding and helped them to understand. A 34-year-old mother of three children observed:

*"...I could understand what she taught me. She taught me how to breastfeed my baby, not to give any water or anything else after birth but breastfeed only; with other children I used to give some other things before my breast but when she taught me, I changed my practice and I did not give my baby anything except the breast"*.

Some women even related the well-being of their babies to the way the peer counsellors had explained about exclusive breastfeeding: An 18-year-old mother of two children said:

*"...I understood what the peer counsellor was teaching me and because I practised what she taught me, my baby has never fallen sick"*.

Despite having breastfed their earlier babies, many said they had never before learned how to hold their babies properly during breastfeeding. A 35-year-old mother of five children said:

*"...She *(peer counsellor) *taught me how to position my baby on the breast, how to put my baby on breast so that all the black part of the breast is in the mouth and when I did it my baby has been breastfeeding and growing well".*

Other women emphasised that with the help of the peer counsellors they had been able to change their breastfeeding practices for the better. A 28-year-old mother of five children said:

*"...Before the peer counsellor taught me, I used to give my babies porridge before one month but this time I did not give porridge early"*.

The pressure to give water or other supplements could be hard to resist and some women felt empowered by the visits to take a firm stand on practising what they had learned from the peer counsellor. An 18-year-old mother of two children observed:

*"...she *(peer counsellor) *told me that when I give birth I should give my baby the breast not water though someone I went with to the health unit wanted me to give water but I refused".*

Some women, however, reported that they were not satisfied with the information and explanations provided by the peer counsellors as they felt those explanations were inadequate. Others related their dissatisfaction to the number of visits being inadequate. A 32-year-old mother of seven children explained:

*"...she came here few times and even what she taught was not much, and sometimes she would come here when I was not at home"*.

Also, some women explained that their own state of mind made them less receptive to the peer counsellors' teachings. Some had social problems and were too preoccupied with those to listen to the peer counsellors. A 25-year-old mother of two children simply said:

"...I was stressed as I had many problems disturbing me. I could not pay a lot of attention to the peer counsellor".

### A useful service

Almost all the women (97%) felt that the peer counselling visits were useful (Table 1). The women gave a variety of reasons, the main ones being: the baby benefited from the peer counsellor's teaching, the woman acquired knowledge about breastfeeding, and she managed to breastfeed exclusively for six months and learned to position her baby properly on the breast. In addition to these benefits, some women said that by breastfeeding exclusively, they saved money that would have been spent on buying cow's milk and sugar.

Some women viewed the usefulness of the peer counsellor visits by referring to the benefits they got themselves. A 35-year-old mother of six children observed:

*"...the peer counsellor taught me during pregnancy and she came back after delivery when my breasts were very full, painful, and swollen, and then she helped me to express some breast milk and I felt relieved"*.

Some women who were having their first breastfeeding experience felt the peer counsellor's visits helped them to cope with a new experience. A 20-year-old mother of one child said:

*"...the peer counsellor showed me how to put my baby on my breast properly and since it was the first experience for me, it was useful"*.

The few women who felt the visits were not useful gave reasons such as: a feeling that she was taught nothing, what she was taught was useless, and her baby was not being satisfied with breast milk alone.

More than half the women (56%) felt there was nothing they wanted to know about breastfeeding that the peer counsellor did not tell them. For the 44% who felt their needs were not met by the peer counsellors, their reasons were further explored. They wanted the peer counsellors to discuss additional issues such as complementary feeding and family planning, which were not part of the peer counselling scheme.

### Interaction between peers

Almost all the women (95%) felt they were able to interact freely with their peer counsellors (Table 1). The good interaction was linked to the approach to the women, being a woman, being familiar and "sitting with" the women during visits, and the content of messages given (Table 3). A 24-year-old mother of four children said:

**Table 3:** Feeling free to discuss with peer counsellor (n = 370, multiple responses allowed for each woman)

Reasons why mother felt free to discuss with peer counsellor*	n	%
I wanted to learn more from the peer counsellor	117	32

The peer counsellor was known to me as we live in the same village	76	21

She brought us good and important information for children	64	17

She was friendly with a good approach	50	14

She is a fellow woman and a mother	26	7

She found me at home	12	3

The peer counsellor used to visit me when I had no work	12	3

She was a visitor in my home	11	3

She was patient with me	10	3

I asked her questions	7	2

Peer counsellor sat with me	6	2

Others	21	6

**Reasons for not feeling free**		

Peer counsellor was new to me	4	1

She was older than me	4	1

I had little time for the peer counsellor	4	1

I was half attentive to her because I had problems	1	0.3

*The reasons are not equal to the number of women interviewed because some women gave more than one reason while others gave none

*"...I knew that she had the knowledge and in case of any problem she could assist me so I didn't see the reason why I should fear her"*,

while a 26-year-old mother of three children said:

*"...she would sit with me and we talked for long about breastfeeding my baby"*.

The women were able to identify with the peer counsellors and they felt this explained why they felt free with them during visits. An 18-year-old mother of one child said:

*"I was free with her because she was a mother like me"*.

The husbands also influenced how free the women felt to discuss with the peer counsellors.

A 42-year-old mother of eight children said:

"...I first informed my husband about it and he allowed me to join the study and I was eager to learn".

On the other hand, the few women who said they did not feel free with the peer counsellor felt she was too old to be a peer to them or was simply a stranger (Table 3).

Almost all the women (99%) said the peer counsellor showed them enough respect (Table 1) by talking to them nicely and politely, asking them for permission before talking to them, going to their homes and answering their questions patiently, greeting them first, addressing them politely - for example calling them "mayi" (mother) - and thanking them for agreeing to talk to her. Other reasons were: knocking on the door and waiting to be allowed inside the house and allowing the woman to complete whatever tasks she was engaged in before discussing with her (Table 4). Most of the reasons given by the women alluded to the approach of the peer counsellor and how she presented herself to the women during visits.

**Table 4:** Mothers feeling respected by peer counsellor (n = 370, more than one response allowed for each woman)

Reasons why mother felt she was given respect	n	%
Peer counsellor talked to me nicely and politely	141	38

She first asked my permission before talking to me	90	24

She came to my home as a visitor	70	19

She was patient and answered my questions	38	10

She would greet me and greet my husband	22	6

She called me "mayi" (=mother)	19	5

She would thank me for allowing me to talk to her	17	5

She knocked on my door and waited humbly	13	4

She would let me finish whatever I was doing before we talk	12	3

She sat next to me	12	3

She is good and well behaved in the community	11	3

She does not minimise me	10	3

She would take the seat I offered her	10	3

Others	47	13

**Reasons why mother felt she was not given respect**		

Because I wasn't the same age as her	1	0.3

*The reasons are not equal to the number of women interviewed because some women gave more than one reason while others gave none

The women were asked how they felt about the whole process of being visited by peer counsellors. The main responses revolved around having acquired knowledge, having been encouraged by the usefulness of the counselling, and their baby having benefited from the whole exercise. The less frequently mentioned reasons included being happy to be taught at home for free and even getting other forms of support such as financial assistance from the peer counsellor.

On the other hand, some women had negative feelings, such as the peer counsellor not giving them enough information about how to take care of their babies, and not understanding most of the things that were taught. One woman felt she was given only one visit, which was not enough, and two women had excessively crying babies, which they attributed to not being satisfied with the breast milk alone.

### Future peer counselling

Almost all the women (98%) would welcome a peer counsellor for their next lactation. The main reasons they gave for this were a desire to learn more about feeding their babies as the peer counsellor might come back with new ideas and that peer counsellors' teaching was important for children's lives, while some felt they needed reminding in case they forgot what they had been taught. Some referred to the peer counsellor's attributes of teaching well and having a good approach. A minority of women, who felt they would not need a peer counsellor for their next lactation, said they had already learned enough about breastfeeding.

Almost all the women said they would recommend a peer counsellor for their friend, and gave the following main reasons: wanting their friend to learn how to feed babies and for their friend to have healthy babies as a result of the peer counselling. However, one woman said the peer counsellor had become her friend and she did not want to lose her friendship. One woman felt that she had learned enough from the peer counsellor to act as a peer counsellor herself. She said she would not refer her friend to a peer counsellor, but would instead first counsel her and only refer her if she met difficulties she could not handle.

Most of the women (71%) considered the peer counsellor the best category of person for helping women with breastfeeding, while 16% preferred a health worker. The main reasons given by the women for preferring a peer counsellor included her being friendly with a good approach while visiting them, having been trained and with good ideas, and the fact that no one had provided teaching before the peer counsellor (Table 5). Those who preferred a health worker attributed their preference to the health worker being trained and experienced in health matters (Table 5).

**Table 5:** Preference of mothers regarding peer counselling (n = 370, more than one response allowed for each woman)

Reasons for preferring peer counsellor	n	%
She is friendly, handles us well, asks for my consent before teaching me and is known to us	137	37

Nobody else has been doing it apart from peer counsellor	61	17

She is trained, has good ideas and I believe what she teaches	57	15

She lives in our community and is easily accessible for assistance	53	14

She comes to my home	32	9

She is respectful and accommodative despite your status	11	3

Others	2	1

**Reasons for preferring others**		

The health worker is trained and has more knowledge/experience	49	13

Friend is near us, emphasise breastfeeding and love children	7	2

Traditional birth attendants have been trained and they know something about delivery and breastfeeding	8	2

Grandmothers have experience since they have given birth and are older than us	6	2

Others	22	6

*The total number of reasons is not equal to the number of women interviewed because some women gave more than one reason while others gave none

## Discussion

In this study we evaluated women's experiences of peer counselling for exclusive breastfeeding. The vast majority of women were very positive about peer counselling. The few who expressed negative feelings did not discredit peer counselling as such, but rather were dissatisfied with specific aspects of the counselling they received, for example having received too few visits or inadequate information.

The women who had received at least the stipulated five visits were more satisfied with the peer counselling. Most of the women expressed general satisfaction with the time the peer counsellor spent with them and considered the visits useful. They felt they were able to interact freely with the peer counsellors. The women identified needs that were not met by the peer counsellors as mainly knowledge about complementary feeding and family planning, which had not been specifically targeted by the intervention.

Exploring satisfaction with care often presents methodological challenges. When and where the evaluation is undertaken may influence client response and some may give socially desirable answers. Using more than one interviewer may introduce some inter-observer errors. In this round of exit interviews we tried to minimise this type of bias by training the research assistant who carried out the interviews and stressing the importance of recording the women's responses to questions verbatim. The research assistant explained to the mothers the importance of ascertaining their true feelings about the peer counselling as it would help to improve it in the future. JN was assisted during the interviews with the women by an interpreter who was not part of the intervention team. The responses to the open-ended questions were coded by a team that included members not directly involved in the intervention.

The time between the peer counsellor visits and the interviews was different for the different mothers, and long periods between peer counselling and interviews may have introduced recall bias. In an effort to minimise this bias, the interviews were conducted soon after the women completed the peer counsellor schedule. Where the woman was not found at home, the research assistant kept returning to the home for the interview until she was found or confirmed as lost to follow-up. In this study the mothers received multiple visits from the peer counsellors and this could stimulate them to remember more than if they were visited only once. Using both open-ended and closed-ended questions could have helped stimulate the women to remember more about their experiences with the peer counsellors. A large number of women participated in these interviews and this increased the chances of establishing a more realistic picture of the participating women's experiences.

A number of factors may have influenced the women's positive experiences with the peer counselling intervention. The time the women spent with the peer counsellors could be viewed in terms of the number of visits each one received as well as the quality of the visits. The women who received more visits expressed greater satisfaction with the different aspects of the peer counselling. It is possible that with more visits the rapport between the peer counsellor and the woman improved, giving the woman the opportunity to learn more and ask for clarification of any unclear messages. A similar finding was previously reported in Toronto, Canada, where the total number of contacts between the peer volunteer and the women, as well as the length of the peer volunteer relationship, correlated with the mothers' evaluation of their peer support experience [29].

Furthermore, the peer counsellor's attitude during each visit could have influenced the woman's experience. This may be especially true if the peer counsellor showed the woman that she had time to discuss with her and address all her concerns. A peer counsellor who seems to be in a hurry may make a woman feel less free to discuss her issues and ask questions. The woman might feel she was burdening the counsellor by asking questions. This may lead to the woman feeling she was not given enough time by the peer counsellor. In this study, many women reported that the peer counsellor took time with them and did not appear to be in a hurry and this could have been responsible for the women's positive attitudes.

It is noteworthy, however, that the issue of "enough time" may be relative as it may be influenced by a woman's understanding of the issue under discussion. One who understands well an issue she wants to discuss might consider the time spent as "enough" simply because her concerns have been addressed to her satisfaction. Furthermore, the cultural context of the study needs to be understood. Since visitors to a home are treated well, the women could have felt obliged to say positive things about the peer counsellors whom they treated as visitors to their homes.

The women generally felt that the peer counsellor visits were useful to them. Some even felt that peer counselling had empowered them to make decisions about how to feed their babies since they now had the knowledge. This is important, as it implies that peer counsellors have to be well prepared through training in order to help the women feel empowered to make decisions about feeding their infants. The benefit to the babies from the visits was echoed by many women and this could have influenced how the women felt towards the intervention. Similar findings have been reported in high-income countries [22,23,35] and in Asia [16] where women expressed a feeling of being empowered to breastfeed their babies.

There were a number of components to the interaction between the peer counsellors and women, such as time spent and social aspects of the relationship. Women highlighted issues of trust, identifying with the peer counsellors, as factors that may have facilitated free discussion and better understanding. The approach of the peer counsellors was highlighted as important as they were able to sit with the women and discuss breastfeeding issues as equals. This reiterates what was reported in the United Kingdom, where support workers had time to sit with the women and observe them, an action that was valued by the women [30]. This further highlights the concept of "peer" to these women and how peer counselling is appreciated by them depending on how it is packaged. It may be important to consider who appropriate peers in different communities are. The importance of the social aspects of peer counselling that are highlighted in the current study was noted in earlier studies [30]. In Bangladesh, certain attributes of the peer counsellors, such as their occupation or trade, made them more acceptable to the mothers [36].

The issue of the women feeling respected by the peer counsellors is important as it may affect the success of the intervention. Showing respect is very much about general politeness as defined in a certain cultural setting. In this study setting, going to someone's home may reflect acceptance on the part of the visitor, so the women could have considered the peer counsellor visits as a sign of being accepted by them. This may have influenced how the women received the messages brought by the peer counsellors. However, as much as visitors are valued and respected, they are also expected to show respect to their hosts. The issue of age seems to be related to respect as the woman who felt she was not respected by the peer counsellor attributed it to the age difference between them. Older women may not respect the views of younger ones, or younger women might just feel that older ones do not respect their opinions. This is rooted in most cultures where increasing age is believed to be associated with more wisdom and older persons expect to be respected because of their age. This may be an important aspect to consider when identifying appropriate peers to allow free interaction with the women.

More than half the women felt there was nothing they missed learning from the peer counsellors, a finding similar to that reported in Toronto, Canada, where most mothers felt there was nothing they would have wanted the peer counsellor to do differently [29]. However, the women who had needs that the peer counselling did not meet identified complementary feeding and family planning as areas about which they wished to learn more. This is not surprising as the current intervention focused mainly on exclusive breastfeeding for the first six months of life, but the women were concerned about what to feed their babies after the age of six months and how to gain control over their fertility. This highlights the importance of planning a complete infant feeding package that covers exclusive breastfeeding, complementary feeding and possibly mothers' health. If the scope of their counselling activities is widened, the challenge will be how much information the peer counsellors can handle in the short training period in order to be able to provide adequate explanations to the women. This finding also supports the argument for integrating breastfeeding promotion into wider child and maternal health interventions, an approach that has been well discussed in an earlier publication [37].

The women generally had positive feelings about the peer counselling process. Acquisition of knowledge and benefits to the baby were highlighted by the women as important explanations for their positive responses. This is similar to previous reports in which women appreciated peer counsellors' support towards successful exclusive breastfeeding [16,23,28]. The women who expressed negative feelings about the intervention voiced concerns about having received insufficient information about breastfeeding or having received few visits, rather than being generally negative. Their concern seemed to be related to how the peer counselling was presented to them rather than not wanting peer counselling at all, and their comments could be used to improve the intervention. Similar sentiments were expressed by some Bangladeshi women, who complained that though some peer counsellors could deliver the messages about breastfeeding, they could not give them adequate explanations, hence leaving them unconvinced [36].

One of the less frequently given reasons for having positive feelings towards the peer counselling process was women having received some form of financial assistance from the peer counsellor. This was a rarely practised gesture by the peer counsellors and was not encouraged by the study team. That notwithstanding, there were reported instances where a peer counsellor encountered a desperate situation during a visit where a young mother had been abandoned by her husband with no support and she felt compelled to give some money to buy soap for washing the baby's clothes. Such instances highlight some of the dilemmas faced by the peer counsellors as they visited the women to help them with breastfeeding.

Almost all the women expressed a desire to have a peer counsellor for their next pregnancy and felt they would recommend a peer counsellor for a friend. The positive effect they felt the peer counselling for breastfeeding had on their babies' health might have influenced this. Similar sentiments were expressed by mothers in Canada, where 85% of the supported women stated they would have a peer volunteer again and suggested that every new mother should be offered peer counselling [29].

Most of the supported women preferred a peer counsellor to a health worker. The reasons they gave for this preference were related to the peer counsellor's good approach to them during visits and the fact that nobody had helped them with breastfeeding before. Similar findings were reported in earlier studies, where supported women favoured a peer counsellor, who they considered to have helped them more than any other people they knew [35] and to have been their most important source of infant feeding advice [22]. The issues of familiarity, availability and living in the same community and hence easy accessibility are important points raised by the women, and could be considered in future planning of similar interventions. However, for those who preferred a health worker, their concern was mainly the conviction that the health workers were well trained for the job.

## Conclusion

The majority of the women were positive towards the process of peer counselling. They were highly appreciative of the individual peer counselling approach for support of exclusive breastfeeding, so this approach could prove useful in promoting and demystifying exclusive breastfeeding in similar settings where it is rarely practised.

## Competing interests

The authors declare that they have no competing interests.

## Authors' contributions

All authors participated in the design and planning of the study; the field work was conducted by JN and VN, supported by JKT; the analysis and write-up was done mainly by JN and TT. All authors read and approved the final manuscript.

## References

[B1] Victora CG Smith PG Vaughan JP Nobre LC Lombardi C Teixeira AM Fuchs SM Moreira LB Gigante LP Barros FC Evidence for protection by breastfeeding against infant deaths from infectious diseases in Brazil Lancet 1987 2 319 322 10.1016/S0140-6736(87)90902-0 2886775

[B2] Jones G Steketee RW Black RE Bhutta ZA Morris SS How many child deaths can we prevent this year? Lancet 2003 362 65 71 10.1016/S0140-6736(03)13811-1 12853204

[B3] Ssenyonga R Muwonge R Nankya I Towards a Better Understanding of Exclusive Breastfeeding in the Era of HIV/AIDS: A Study of Prevalence and Factors Associated with Exclusive Breastfeeding from Birth, in Rakai, Uganda J Trop Pediatr 2004 50 348 353 10.1093/tropej/50.6.348 15537720

[B4] Semega-Janneh IJ Bohler E Holm H Matheson I Holmboe-Ottesen G Promoting breastfeeding in rural Gambia: combining traditional and modern knowledge Health Policy Plan 2001 16 199 205 10.1093/heapol/16.2.199 11358922

[B5] Nwankwo BO Brieger WR Exclusive breastfeeding is undermined by use of other liquids in rural southwestern Nigeria J Trop Pediatr 2002 48 109 112 10.1093/tropej/48.2.109 12022424

[B6] Engebretsen IM Wamani H Karamagi C Semiyaga N Tumwine J Tylleskar T Low adherence to exclusive breastfeeding in Eastern Uganda: a community-based cross-sectional study comparing dietary recall since birth with 24-hour recall BMC Pediatr 2007 7 10 10.1186/1471-2431-7-10 17331251 PMC1828054

[B7] Black RE Allen LH Bhutta ZA Caulfield LE de Onis M Ezzati M Mathers C Rivera J Maternal and child undernutrition: global and regional exposures and health consequences Lancet 2008 371 243 260 10.1016/S0140-6736(07)61690-0 18207566

[B8] Mukasa GK A 12-month lactation clinic experience in Uganda J Trop Pediatr 1992 38 78 82 1569640 10.1093/tropej/38.2.78

[B9] Poggensee G Schulze K Moneta I Mbezi P Baryomunsi C Harms G Infant feeding practices in western Tanzania and Uganda: implications for infant feeding recommendations for HIV-infected mothers Trop Med Int Health 2004 9 477 485 10.1111/j.1365-3156.2004.01214.x 15078266

[B10] Wamani H Astrom AN Peterson S Tylleskar T Tumwine JK Infant and young child feeding in western Uganda: knowledge, practices and socio-economic correlates J Trop Pediatr 2005 51 356 361 10.1093/tropej/fmi048 15947011

[B11] Iliff PJ Piwoz EG Tavengwa NV Zunguza CD Marinda ET Nathoo KJ Moulton LH Ward BJ Humphrey JH Early exclusive breastfeeding reduces the risk of postnatal HIV-1 transmission and increases HIV-free survival AIDS 2005 19 699 708 10.1097/01.aids.0000166093.16446.c9 15821396

[B12] Coutsoudis A Pillay K Kuhn L Spooner E Tsai WY Coovadia HM Method of feeding and transmission of HIV-1 from mothers to children by 15 months of age: prospective cohort study from Durban, South Africa Aids 2001 15 379 387 10.1097/00002030-200102160-00011 11273218

[B13] Coutsoudis A Kuhn L Pillay K Coovadia HM Exclusive breast-feeding and HIV transmission Aids 2002 16 498 499 10.1097/00002030-200202150-00028 11834968

[B14] Coovadia HM Rollins NC Bland RM Little K Coutsoudis A Bennish ML Newell ML Mother-to-child transmission of HIV-1 infection during exclusive breastfeeding in the first 6 months of life: an intervention cohort study Lancet 2007 369 1107 1116 10.1016/S0140-6736(07)60283-9 17398310

[B15] WHO Consensus Statement from HIV and infant feeding technical consultation, Geneva 2006

[B16] Haider R Ashworth A Kabir I Huttly SR Effect of community-based peer counsellors on exclusive breastfeeding practices in Dhaka, Bangladesh: a randomised controlled trial Lancet 2000 356 1643 1647 10.1016/S0140-6736(00)03159-7 11089824

[B17] Hoddinott P Chalmers M Pill R One-to-one or group-based peer support for breastfeeding? Women's perceptions of a breastfeeding peer coaching intervention Birth 2006 33 139 146 10.1111/j.0730-7659.2006.00092.x 16732780

[B18] Hodnett E Efficacy of home-based peer counseling to promote exclusive breast-feeding: a randomized controlled trial J Pediatr 1999 135 649 650 10577054

[B19] Kistin N Abramson R Dublin P Effect of peer counselors on breastfeeding initiation, exclusivity, and duration among low-income urban women J Hum Lact 1994 10 11 15 10.1177/089033449401000121 7619241

[B20] Kramer MS Chalmers B Hodnett ED Sevkovskaya Z Dzikovich I Shapiro S Collet JP Vanilovich I Mezen I Ducruet T Promotion of Breastfeeding Intervention Trial (PROBIT): a randomized trial in the Republic of Belarus Jama 2001 285 413 420 10.1001/jama.285.4.413 11242425

[B21] McInnes RJ Love JG Stone DH Evaluation of a community-based intervention to increase breastfeeding prevalence J Public Health Med 2000 22 138 145 10.1093/pubmed/22.2.138 10912550

[B22] Morrow AL Guerrero ML Shults J Calva JJ Lutter C Bravo J Ruiz-Palacios G Morrow RC Butterfoss FD Efficacy of home-based peer counselling to promote exclusive breastfeeding: a randomised controlled trial Lancet 1999 353 1226 1231 10.1016/S0140-6736(98)08037-4 10217083

[B23] Muirhead PE Butcher G Rankin J Munley A The effect of a programme of organised and supervised peer support on the initiation and duration of breastfeeding: a randomised trial Br J Gen Pract 2006 56 191 197 16536959 PMC1828262

[B24] Bland RM Little KE Coovadia HM Coutsoudis A Rollins NC Newell ML Intervention to promote exclusive breast-feeding for the first 6 months of life in a high HIV prevalence area AIDS 2008 22 883 891 10.1097/QAD.0b013e3282f768de 18427207

[B25] Raine P Promoting breast-feeding in a deprived area: the influence of a peer support initiative Health Soc Care Community 2003 11 463 469 10.1046/j.1365-2524.2003.00449.x 14629576

[B26] Schafer E Vogel MK Viegas S Hausafus C Volunteer peer counselors increase breastfeeding duration among rural low-income women Birth 1998 25 101 106 10.1046/j.1523-536x.1998.00101.x 9668744

[B27] Shaw E Kaczorowski J The effect of a peer counseling program on breastfeeding initiation and longevity in a low-income rural population J Hum Lact 1999 15 19 25 10.1177/089033449901500108 10578771

[B28] Ingram J Rosser J Jackson D Breastfeeding peer supporters and a community support group: evaluating their effectiveness Matern Child Nutr 2005 1 111 118 10.1111/j.1740-8709.2005.00005.x 16881886 PMC6860964

[B29] Dennis CL Breastfeeding peer support: maternal and volunteer perceptions from a randomized controlled trial Birth 2002 29 169 176 10.1046/j.1523-536X.2002.00184.x 12153647

[B30] Beake S McCourt C Rowan C Taylor J Evaluation of the use of health care assistants to support disadvantaged women breastfeeding in the community Matern Child Nutr 2005 1 32 43 10.1111/j.1740-8709.2004.00007.x 16881877 PMC6874387

[B31] UBOS Uganda Population and Housing Census 2002 Uganda Bureau of Statistics (UBOS)

[B32] Johnson RB Onwuegbuzie AJ Mixed methods research: a research paradigm whose time has come Educational Researcher 2004 33 14 26 10.3102/0013189X033007014

[B33] Teddlie C Tashakkori A Tashakkori A, Teddlie C Major issues and controversies in the use of mixed methods in the social and behavioural sciences Handbook of Mixed Methods in Social and Behavioral Research 2002 Sage 3 50

[B34] Nankunda J Tylleskar T Ndeezi G Semiyaga N Tumwine JK Establishing individual peer counselling for exclusive breastfeeding in Uganda: implications for scaling-up Matern Child Nutr 2010 6 53 66 10.1111/j.1740-8709.2009.00187.x 20055930 PMC6860637

[B35] Martens PJ Increasing breastfeeding initiation and duration at a community level: an evaluation of Sagkeeng First Nation's community health nurse and peer counselor programs J Hum Lact 2002 18 236 246 12192958 10.1177/089033440201800305

[B36] Haider R Kabir I Huttly SR Ashworth A Training peer counselors to promote and support exclusive breastfeeding in Bangladesh J Hum Lact 2002 18 7 12 10.1177/089033440201800102 11845742

[B37] Labbok MH Transdisciplinary breastfeeding support: creating program and policy synergy across the reproductive continuum Int Breastfeed J 2008 3 16 10.1186/1746-4358-3-16 18680583 PMC2538507

